# Biochemical, Kinetic, and Spectroscopic Characterization of *Ruegeria pomeroyi* DddW—A Mononuclear Iron-Dependent DMSP Lyase

**DOI:** 10.1371/journal.pone.0127288

**Published:** 2015-05-19

**Authors:** Adam E. Brummett, Nicholas J. Schnicker, Alexander Crider, Jonathan D. Todd, Mishtu Dey

**Affiliations:** 1 Department of Chemistry, University of Iowa, Iowa City, Iowa, United States of America; 2 School of Biological Sciences, University of East Anglia, Norwich Research Park, United Kingdom; United States Army Medical Research Institute of Infectious Diseases, UNITED STATES

## Abstract

The osmolyte dimethylsulfoniopropionate (DMSP) is a key nutrient in marine environments and its catabolism by bacteria through enzymes known as DMSP lyases generates dimethylsulfide (DMS), a gas of importance in climate regulation, the sulfur cycle, and signaling to higher organisms. Despite the environmental significance of DMSP lyases, little is known about how they function at the mechanistic level. In this study we biochemically characterize DddW, a DMSP lyase from the model roseobacter *Ruegeria pomeroyi* DSS-3. DddW is a 16.9 kDa enzyme that contains a C-terminal cupin domain and liberates acrylate, a proton, and DMS from the DMSP substrate. Our studies show that as-purified DddW is a metalloenzyme, like the DddQ and DddP DMSP lyases, but contains an iron cofactor. The metal cofactor is essential for DddW DMSP lyase activity since addition of the metal chelator EDTA abolishes its enzymatic activity, as do substitution mutations of key metal-binding residues in the cupin motif (His81, His83, Glu87, and His121). Measurements of metal binding affinity and catalytic activity indicate that Fe(II) is most likely the preferred catalytic metal ion with a nanomolar binding affinity. Stoichiometry studies suggest DddW requires one Fe(II) per monomer. Electronic absorption and electron paramagnetic resonance (EPR) studies show an interaction between NO and Fe(II)-DddW, with NO binding to the EPR silent Fe(II) site giving rise to an EPR active species (g = 4.29, 3.95, 2.00). The change in the rhombicity of the EPR signal is observed in the presence of DMSP, indicating that substrate binds to the iron site without displacing bound NO. This work provides insight into the mechanism of DMSP cleavage catalyzed by DddW.

## Introduction

The osmolyte molecule dimethylsulfoniopropionate (DMSP) is one of Earth’s most abundant organosulfur molecules with ∼10^9^ tons per annum, being made worldwide by many marine phytoplankton (coccolithophores, dinoflagellates and diatoms), macroalgae, a few angiosperms and some corals [1], [2], [3]. DMSP can occur at remarkable intracellular levels (as high as 0.5 M) in these eukaryotic organisms. Many of these organisms, e.g. *Emiliania huxleyi*, form blooms, which, when lysed by viruses, result in large inputs of DMSP into the marine milieu with important environmental consequences due to its catabolism by microorganisms [4]. Although some marine eukaryotic organisms have been shown to catabolize DMSP, e.g. *E*. *huxleyi*, the majority is thought to be carried out by bacteria through either the DMSP demethylation or DMSP lyase pathways [5], [6].

**Fig 1:**
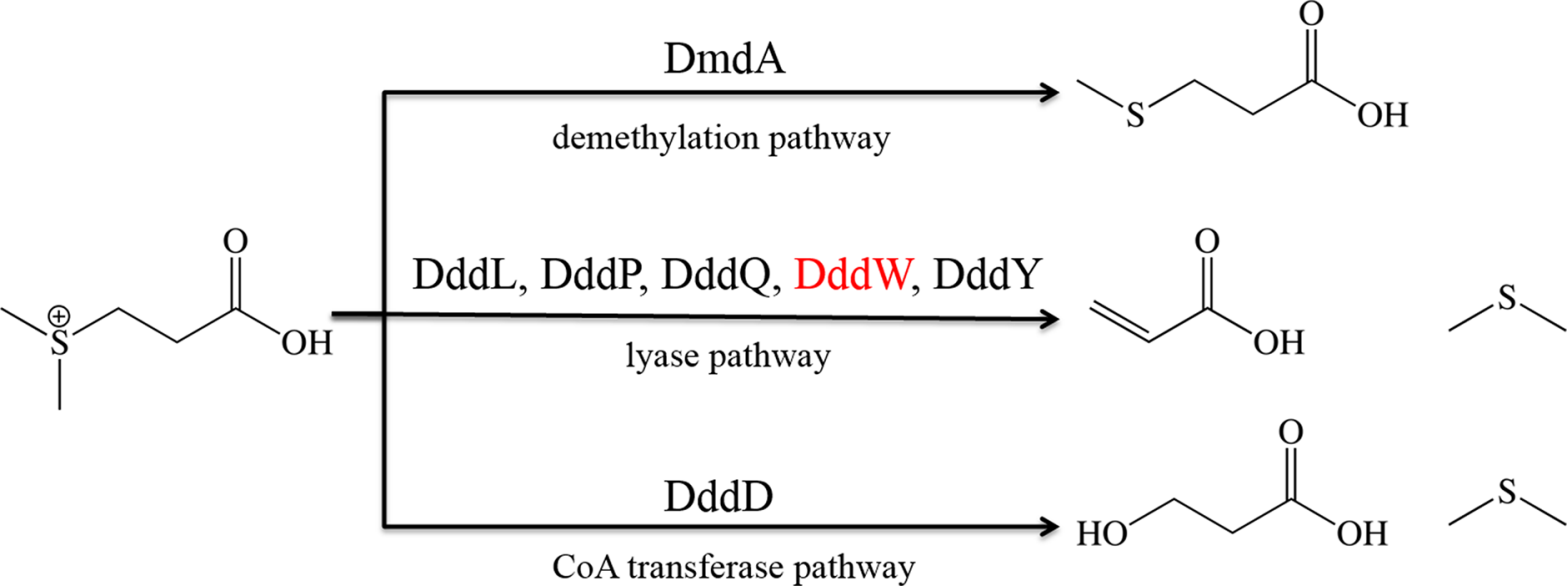
Pathways for DMSP biodegradation. The DmdA enzyme *via* the demethylation pathway (top) removes a methyl group from DMSP generating methylmercaptopropionate (MMPA). The DddL, DddP, DddQ, DddW and DddY DMSP lyase enzymes produces acrylate and DMS from DMSP via the typical DMSP lyase pathway (middle). The cupin containing DddW (shown in red) is investigated here to gain insight into the mechanism of thioether bond cleavage of DMSP. An atypical DMSP lyase DddD generates DMS and 3-hydroxy-propionate *via* a CoA transferase pathway (bottom).

It is believed that the global majority of DMSP is degraded through the demethylation pathway by abundant marine α-proteobacteria known as the Roseobacters and the ubiquitous SAR11 lineage that contain the *dmdA* gene encoding DMSP demethylase [5], [7], [8]. The demethylation pathway generates methylmercaptopropionate (MMPA), and major downstream catabolites, methanethiol (MeSH) and acetaldehyde [9]. In comparison, the DMSP lyase pathway functions in a far broader range of microorganisms, including bacteria, eukaryotic phytoplankton, macroalgae, and fungi, and generates volatile dimethylsulfide (DMS) together with the catabolites, acrylate or 3-hydroxypropionate through elimination reactions (Fig 1) [5], [10]. DMS generated by this pathway serves as a key nutrient for marine bacteria [10], but more significantly it is the most abundant sulfur compound emitted to the atmosphere [11]. Atmospheric DMS is oxidized to create aerosols that act as cloud condensation nuclei affecting climate and are key in biogeochemical cycling of sulfur [10], [12]. DMS is also an important signaling molecule studied in marine ecology [13–16], has wide industrial uses [17], at pathologically high concentrations it is associated with blood-borne halitosis [18], [19], and is a component of the smell of the seaside.

Despite the environmental, industrial, and pathological implications of DMS, only relatively recently has genetics been used to identify the genes involved in DMSP dependent DMS production [5]. Six distinct DMSP lyase enzymes occur in bacteria and fungi that are encoded by different *ddd* genes [5]. The first identified DMSP lyase was DddD found mainly in marine γ-proteobacteria and in some α- and β-proteobacteria likely through horizontal gene transfer (HGT) [20], [21], [22]. DddD is a class III CoA-transferase that generates 3-hydroxypropionate, acetate, and DMS as major products using DMSP and acetyl-CoA (Fig 1) [20], [21], [23]. Kinetic and mutagenic studies have shown that a catalytic aspartate in DddD is required for CoA transfer and DMSP lyase activity [23]. Although the detailed catalytic mechanism of DddD has not been elucidated, biochemical data indicates the bi-functionality of DddD, *i*.*e*., CoA transferase and lyase forming 3-hydroxypropionate-CoA and DMS respectively [23]. In contrast, the other five known DMSP lyases (DddL, DddP, DddQ, DddW and DddY) cleave DMSP into acrylate, DMS, and a proton (Fig 1) [5]. The DMSP lyase DddY functions in some β-, ɛ-, and γ-proteobacteria and unlike other lyases it has no known conserved protein domains and exists in the periplasm of bacteria [24]. The remaining four DMSP lyases DddL, DddP, DddQ, and DddW are predicted by the nature of their protein families to require metal cofactors [5], [25], [26]. DddP is a M24B metallopeptidase family enzyme that functions in many strains of marine α -proteobacteria, certain γ-proteobacteria, and in some eukaryotic ascomycete fungi [22], [27], [28]. The recently published crystal structure of DddP from *Roseobacter denitrificans* shows it to be a homodimeric metalloprotein, with a binuclear center of two metal ions in close proximity (2.7 Å) in its active site [26]. Total reflection X-ray fluorescence together with inductively coupled plasma mass spectrometric data suggested that various divalent transition metal ions were tightly bound to DddP, but iron was the most abundant ion present in the protein.

**Fig 2:**
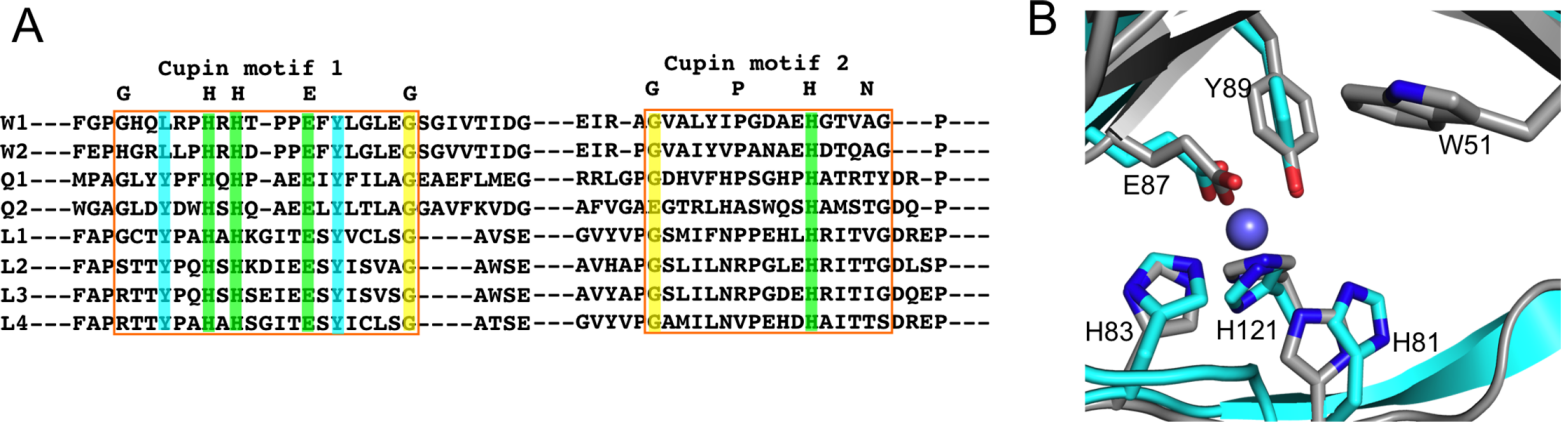
Cupin motifs and metal binding residues of DddW. (A) Sequence alignment of cupin regions of selected DddW, DddQ and DddL proteins using sequences deposited at NCBI and CLUSTAL 2.1 for the alignment. The two conserved cupin motifs 1 (GX_5_HXHX_3,4_EX_6_G) and 2 (GX_5_PXGX_2_HX_3_N), containing residues that bind metal ions and are catalytically important are highlighted in green. Tyr residues playing catalytic role in *Ruegeria lacuscaerulensis* DddQ are marked cyan and other conserved residues in the cupin motifs are colored yellow. The sequences are from: W1 = DddW, *Ruegeria pomeroyi* DSS-3 (SPO0453); W2 = DddW, *Roseobacter sp*. MED193, (MED193_09710); Q1 = DddQ, *Ruegeria pomeroyi* DSS-3 (SPO1596); Q2 = DddQ, *Ruegeria lacuscaerulensis* (ITI-1157); L1 = DddL, *Sulfitobacter sp*. EE-36 (EE36_11918); L2 = DddL, *Rhodobacter sphaeroides* 2.4.1 (RSP_1433); L3 = DddL, *Roseibacterium elongatum* DSM 19469 (roselon_02436); L4 = DddL, *Caenispirillum salinarum* (C882_2645). (B) Homology model of *Ruegeria pomeroyi* DddW (grey) (generated using Phyre 2 [52]) superimposed on the Zn(II)-bound structure of *Ruegeria lacuscaerulensis* DddQ (cyan) (PDB 4LA2). The homology model of DddW shows the catalytic residues H81, H83, E87, and H121. Most of these residues of DddW (H83, E87, and H121) superimpose well on the zinc-coordinating DddQ residues (H125, E129, and H163). While Tyr usually is not involved in metal ion binding in cupin proteins, the DddQ structure shows a Zn-coordinated Tyr residue (Tyr131) and this Tyr superimposes on Tyr89 of DddW. The side chain residues are shown in ball and stick with oxygens in red, nitrogens in blue, zinc in slate, and carbons are similar to protein backbone.

Interestingly, the polypeptide sequence of the remaining three DMSP cleavage enzymes DddL, DddQ, and DddW all contain a C-terminal cupin domain characterized by a β-barrel structure, but have no significant extended sequence identity to each other [29–31]. More specifically, the two conserved sequence motifs of cupins, GX_5_HXHX_3,4_E (or D)X_6_G and GX_5_PXGX_2_HX_3_N, known to contain residues that bind metal ions and play a functional role are present in DddL, DddQ, and DddW [29], [30] (Fig 2A). Various combinations of histidines and one glutamate/aspartate from the two motifs comprise the active site and are involved in binding a metal ion [29–32]. While divalent transition metal ions typically bind at the active site in a cupin-domain containing protein, often these enzymes are promiscuous and accommodate different metal ions [29], [30]. However, commonly there is only one preferred catalytic metal ion that is involved in substrate positioning [29–31]. The first cupin containing DMSP lyase identified was DddL that is found mainly in marine α-proteobacteria [33], but as of yet the enzyme has not been purified and characterized *in vitro*. Recent structural studies on the *Ruegeria lacuscaerulensis* DMSP lyase DddQ reveal that the as-purified DddQ contains a zinc ion in its active site [25]. However, biochemical characterization suggests that zinc, copper, and alkaline earth metal ions inhibit the lyase activity of DddQ, whilst Co(II) and Mn(II) enhance the activity [25]. The structural and biochemical data indicate that DddQ adopts a β-barrel fold with various residues (Tyr120, His123, His125, Glu129, Tyr131, His163) of the cupin motifs being both located in the DddQ active site and essential for catalysis (Fig 2A and 2B) [25].

The focus of this study is on the DMSP lyase DddW, which was isolated as the major DMSP induced gene in the model roseobacter *Ruegeria pomeroyi*, and its expression is controlled by a divergently transcribed LysR-type transcription regulator [34]. This genetic characterization of the *dddW* gene is the only report of the lyase and no biochemical data exists for the isolated enzyme. Since DddW possesses conserved cupin motifs and metal binding residues, as present in DddQ, we set about a biochemical study to understand the mechanism of DMS liberation *via* the DMSP lyase DddW and to investigate the catalytic role of metal ions in this reaction. We overexpressed and purified *R*. *pomeroyi* DddW from *E*. *coli* and explored its metal binding and catalytic characteristics. From detailed kinetic, fluorescence, UV-visible, and EPR spectroscopic studies, we have determined the metal binding and electronic/structural properties of DddW. Using DddW variants, the specific residues (3-His-1-Glu) required for iron binding and catalysis have been identified. Further, the mechanistic implications of the observed binding properties of various metal ions to DddW and their catalytic roles are discussed.

## Materials and Methods

### Materials

All buffers, media ingredients, and other reagents were acquired from Sigma-Aldrich (St. Louis, MO) and unless otherwise stated, were of the highest purity available. Solutions were prepared using Nanopure deionized water. N_2_ (99.98%) and liquid helium (>99%) were obtained from Praxair (Cedar Rapids, IA).

### DMSP Synthesis

DMSP was synthesized from DMS (Alfa Aesar, Ward Hill, MA) and acrylic acid (Alfa Aesar, Ward Hill, MA) using a previous method with minor modifications [20]. A volume of 3.8 mL (52 mmol) DMS was added to a 25 mL solution of 2 M HCl. To this solution, 2.5 g (35 mmol) acrylic acid was added and refluxed for 2 hours. The reaction was cooled to room temperature and evaporated to obtain a thick, colorless oil. The oil was mixed vigorously with 30 mL of a 1:1 ethanol:diethyl ether combination until a white solid was formed. The solid was washed with ethanol and then with diethyl ether to give DMSP in 4.59 g and 98% yield. The purity of the product was routinely checked by ^1^H NMR and melting point analysis.

### The construction of *dddW* expression vectors

The *dddW* gene was amplified from *R*. *pomeroyi* genomic DNA using the primers Wpet1 (AACTGCAGCATATGACCGCCATGCTCGACAGTTTC) and Wpet2 (ATGGATCCTCAGGCGCTGGCGGTGAACCG) and the PCR product was digested with *Nde*I and *Bam*HI and then cloned into the expression vector pET16b (Novagen), forming plasmid pBIO1963, which allows the expression of DddW translationally fused at the N-terminus to 10 histidines. This plasmid was used as the template for site directed mutagenesis with a Quikchange XL kit (Stratagene) according to the manufacturer's instructions. Four site-directed mutants of DddW, namely H81A, H83A, E87A, and H121A were made using mutagenic oligonucleotides yielding the plasmids pBIO2243, pBIO2244, pBIO2245 and pBIO2246 respectively. All plasmid constructs were confirmed by sequencing.

### Growth and expression of recombinant DddW protein

All plasmids were transformed into *E*. *coli* BL21Star DE3 pLysS cells (Life Technologies, Grand Island, NY) for protein expression. *E*.*coli* cells containing pBIO1963 or derivatives were grown in Luria-Bertani broth at 37°C until cultures reached an optical density at 600 nm (OD_600_) of 0.4, at which time the cultures were induced with 0.2 mM isopropyl-D-thiogalactopyranoside (IPTG). The temperature was lowered to 25°C and the cells were grown for 8 hours before harvest. Cells were collected by centrifugation at 6,000 rpm for 30 min at 4°C.

### Growth and expression of DddW in M9 media in the presence of metal ions

*E*.*coli* cells containing pBIO1963 were grown in M9 minimal media in the presence of chloride salts of Mn(II), Fe(III), Co(II), Ni(II), Cu(II), and Zn(II). The cells were grown at 37°C until cultures reached an OD_600_ of 0.5, at which time the corresponding metal chloride was added. Metal ions were added individually and in a separate experiment as a mixture consisting of Mn(II), Fe(III), Co(II), Ni(II), Cu(II), and Zn(II) to final concentrations of 50 μM, 50 μM, 2 μM, 2 μM, 2 μM, and 10 μM respectively. The concentrations were varied between metal ions so as to avoid toxically high concentrations of certain metals. The cultures were grown until OD_600_ of 0.8 and induced with 0.2 mM IPTG. The temperature was then lowered to 25°C and allowed to grow for 12 hours before harvesting.

### Purification and preparation of apo-DddW

The cell pellet from 1 L of culture was re-suspended in 25 mL of lysis buffer (50 mM HEPES pH 7.5, 300 mM NaCl, 5 mM imidazole, 5% glycerol) and placed on ice. Phenylmethylsulfonyl fluoride (2 mM), lysozyme (0.5 mg/mL), one EDTA-free protease inhibitor cocktail tablet (Roche, Indianapolis, IN) per 25 mL lysate, and DNase (5 units) were added and the cell suspension was sonicated for 4 min total time with 5 seconds “on” and 15 seconds “off”. Lysed cells were centrifuged at 30,000 rpm for 30 min at 4°C and soluble fractions containing the protein were used for subsequent purification. The cell extract (25 mL) was applied to a Ni-NTA (5 mL) resin pre-equilibrated with lysis buffer and incubated for one hour at 4°C. The column was washed with 100 mL wash buffer (50 mM HEPES pH 7.5, 300 mM NaCl, 20 mM imidazole, 5% glycerol). The protein was eluted over a gradient between the wash buffer and an elution buffer containing 50 mM HEPES pH 7.5, 300 mM NaCl, 500 mM imidazole, 5% glycerol). The protein purity was determined by SDS-PAGE and pure fractions were pooled, dialyzed five times in 2 L chelation buffer (50 mM MES pH 6.5, 100 mM NaCl, 0.5 mM EDTA, 10% glycerol) to remove protein bound iron. After chelation of excess iron, the sample was concentrated using an Amicon Ultra 5,000 MWCO membrane (Millipore, Billerica, MA) and then exchanged into a storage buffer (50 mM HEPES pH 7.5, 100 mM NaCl, 10% glycerol). Following metal analysis, 100 μL aliquots were prepared and stored at -80°C until use. This purification protocol routinely yields 12–15 mg per liter of purified apo-DddW.

### Reconstitution of DddW

The concentrated apo-DddW was reconstituted as follows. 500 μL of 199 μM apo-protein was incubated with 500 μM Fe(NH_4_)_2_(SO_4_)_2_ for 1 h followed by desalting in a GE PD MiniTrap G-25 column. The column was washed in a buffer containing 50 mM HEPES pH 7.5, 10 mM NaCl. The sample was loaded and allowed to enter the resin before more solution of buffer was added. The reconstituted protein was eluted from the column and 0.5 mL aliquots were collected during the gravity elution. The eluted protein was analyzed for metal content consistently yielding a holo-enzyme with 85–90 mol percent iron.

### Metal Analysis

Protein samples used for metal analysis were in the range of 10–70 μM. Samples were prepared in a solution containing 50% protein in storage buffer, 48% water, and 2% nitric acid. Addition of metal-free nitric acid to the solution denatured the protein, which was removed by centrifugation at 4000 rpm for 15 minutes. The supernatant was used to probe the metal content by inductively coupled plasma optical emission spectrometry (ICP-OES). A blank sample was made with the same composition of storage buffer, water, and nitric acid. Standards of Fe, Mn, Co, Ni, Cu, and Zn (Inorganic Ventures) were used at five different concentrations ranging from 0.18 μM-73 μM.

### Native molecular weight determination by gel filtration and dynamic-light scattering (DLS)

As-isolated apo-DddW (19 mg/mL) in storage buffer was injected onto a Superdex 200 16/600 column (GE Healthcare) pre-equilibrated with the same buffer and eluted at a flow rate of 0.6 mL/min. The following molecular mass standards were used under identical conditions: vitamin B_12_ (1.35 kDa), myoglobin (17 kDa), ovalbumin (44 kDa), γ-globulin (158 kDa), and thyroglobulin (670 kDa). Dynamic light scattering was performed with 58 μM DddW using DynaPro NanoStar (Wyatt Technology, Santa Barbara, CA) equipped with additional static light scattering detector and temperature control.

### Secondary structure determination by circular dichroism (CD)

Protein concentration used for CD measurements was 0.1 mg/mL. Apo-DddW was exchanged into a 10 mM phosphate buffer solution at pH 7.5. The CD spectra were obtained at room temperature in a Jasco J-815 spectropolarimeter (Easton, MD) with a 1 cm path length. Data were collected between 200–250 nm, with a bandwidth of 1 nm, a response time of 2 seconds, and a scan speed of 100 nm/min.

### Fluorescence saturation measurements for metal binding, stoichiometry, and pH dependence

Fluorescence spectra of DddW were recorded at room temperature using a Horiba Jobin Yvon bench-top spectrofluorometer (FluoroMax-4 Horiba Scientific, Edison, NJ). Fluorescence titration was used to determine the binding affinity of different metal ions to apo-DddW. The change of intrinsic protein fluorescence at 295 nm served as an indicator of metal ion binding. Titration experiments were performed using enzyme concentrations ranging from 0.5–2.0 μM in 50 mM HEPES pH 8.0, 20 mM NaCl. All metal binding assays were performed aerobically with the exception of Fe(II) that was prepared anaerobically in a glove box. In a typical anaerobic metal binding assay, the quartz cuvette was thoroughly sealed with a septum and parafilm in order to prevent diffusion of O_2_ into the anaerobic solution and oxidation of Fe(II). Metal salt solutions were added in aliquots of 5–85 μL to a final concentration ranging from 0.005–610 μM depending on the metal ion. At the excitation wavelength of 295 nm the emission signal intensity was measured at the maxima, 330 nm, with both excitation and emission slit widths of 5 nm. Data were fit with the appropriate non-linear regression analysis using SigmaPlot 12.0 (Systat Software Inc., Point Richmond, CA).

The metal ion stoichiometry of DddW was determined by titration of Fe(NH_4_)_2_(SO_4_)_2_ under tight binding conditions and the data was analyzed by nonlinear curve fitting using a reported equation with appropriate modifications [35], [36].

### Activity assays of DddW using HPLC

Unless otherwise noted, all DddW activity assays were performed anaerobically in a Vacuum Atmospheres (Hawthorne, CA) anaerobic chamber maintained under nitrogen gas at <1 ppm of oxygen. The catalytic activity was monitored in an Agilent 1100 series HPLC equipped with single wavelength detector using a Zorbax (Agilent, Santa Clara, CA) 300 SB-C18 column (250 mm × 9.4 mm I.D., 5 μM particle size), which was developed with a 7.5% acetonitrile, 1% phosphoric acid solution, pH 2.0 at a flow rate of 1 mL/min. The assay is based on monitoring the area of absorption signal corresponding to the acrylate product at 205 nm. Activity assays were performed in the presence of various metal ions and the concentration of metal ion was varied so that each metal was in a 90% bound state, based on the K_d_ values. The concentration of DMSP used was 10 mM. The reactions were monitored for 3 mins so as to monitor the linear portion of the rate of formation of acrylate. In a typical assay, 2 μM apo-enzyme in 50 mM HEPES pH 8.0, 20 mM NaCl was mixed with an equimolar concentration of Fe(NH_4_)_2_(SO_4_)_2_ at room temperature. Aliquots were taken at various time points over a period of 3 mins and quenched with 50% phosphoric acid. Denatured protein was removed by centrifugation at 10,000 rpm for 5 minutes and the supernatant was injected into the HPLC for acrylate product analysis.

The pH dependence of activity studies were performed by monitoring the acrylate product. To assess the optimal pH for activity, reactions were performed by adding 10 mM DMSP to an equimolar solution of 2 μM apo-DddW and Fe(NH_4_)_2_(SO_4_) in the following reaction buffers at varying pH: 50 mM MES, 20 mM NaCl (pH 5.5, 6.0, 6.5), 50 mM HEPES, 20 mM NaCl (pH 7.0, 7.5, 8.0), 50 mM Tris-HCl, 20 mM NaCl (pH 8.5, 9.0).

To determine the kinetic parameters of DddW in presence of Fe(II) and Mn(II), the reaction was initiated by adding varying concentrations of DMSP ranging from 0.5 mM to 35 mM. DddW activity determination with mutant enzymes was performed anaerobically following the procedure described above.

### Product identification by liquid chromatography mass spectrometry (LC-MS) analysis

An anaerobic reaction mixture consisting of 2 μM each of apo-DddW and Fe(NH_4_)_2_(SO_4_)_2_, was prepared in 50 mM HEPES pH 8.0, 20 mM NaCl and to this solution 10 mM DMSP was added. The reaction was incubated for 30 minutes, followed by acid quenching and centrifugation to remove denatured protein. The supernatant was analyzed by LC-MS to identify the acrylate product. 10 μL samples were loaded onto an Acquity UPLC BEH C18 (2.1 x 100 mm) column (Waters Corporation, Milford, MA) and ran isocratically at 0.2 mL/min for 25 mins in a mobile phase consisting of 92.4% H_2_O, 7.5% acetonitrile, and 0.1% formic acid. Data was collected in negative electrospray ionization (ESI) mode with a mass range of m/z = 45–600.

### Spectroscopy of DddW

All UV-visible spectra were measured in an anaerobic chamber using an Ocean Optics DH-2000-BAL light source (Ocean Optics, Dunedin, FL). The spectrum of apo-DddW at 370 μM concentration was collected followed by the addition of an equimolar concentration of Fe(NH_4_)_2_(SO_4_)_2_ and the spectrum recorded. The Fe(II)-DddW solution was moved out of the glove box and nitric oxide gas (35 mL) was then slowly bubbled through this solution contained in a sealed vial. The Fe(II)-DddW mixture saturated with NO was subsequently moved back into the anaerobic chamber for UV-visible data collection. For samples with Cu(II), spectra were first recorded with 1 mM apo-DddW followed by the addition of 1 mM copper(II)chloride. All spectra were measured from 200 nm-900 nm.

X-band EPR spectra were recorded on a Bruker EMX spectrometer (Bruker Biospin Corp., Billerica, MA), equipped with an Oxford ITC4 temperature controller and an ESR900 cryostat, a Hewlett-Packard model 5340 automatic frequency counter, and Bruker gaussmeter. The EPR spectroscopic parameters included microwave frequency 9.43 GHz, receiver gain 2 x 10^4^, modulation frequency 100 kHz, 16 scans, sweep time 83.89 s, 200 μW microwave power, 10 G modulation amplitude, and 4 K. The EPR samples were prepared anaerobically with enzyme concentrations in the range of 18–370 μM in 50 mM HEPES pH 7.5, 20 mM NaCl. Reduced Fe(II)-DddW samples were prepared by adding sodium dithionite to a final concentration of 1mM. Addition of NO was achieved by bubbling the gas through an enzyme solution in a sealed vial.

### Data Analysis and Equations

Data were fit with SigmaPlot (Systat Software Inc., Point Richmond, CA). DddW activity assays were fit to a Michaelis-Menten kinetics model. The data for V_max_ and K_m_ were fit to Eq (1)

$$y=\frac{V_{max}\times x}{K_{m}+x}$$ (1)

where y and x in Eq (1) are the reaction rate and substrate concentration respectively, V_max_ is the maximum initial rate of acrylate formation and K_m_ is the concentration of DMSP at which the reaction rate is at half-maximum (Michaelis-Menten constant).

Metal binding affinities were fit to a ligand-binding model. The data for K_d_ were fit to Eq (2) using SigmaPlot

$$y=\frac{Y_{o}-(x\times Y_{max})}{K_{d}+x}$$ (2)

where, x is the concentration of metal ion added and y is the measured fluorescence intensity, Y_o_ is the initial fluorescence intensity, Y_max_ is the fluorescence intensity at maximum saturation, K_d_ is the equilibrium dissociation constant of the binding metal ion and is the concentration at which the binding site is 50% occupied by the respective metal ion. This equation is derived assuming that the concentration of metal ion added (x) is approximately equal to the amount of free metal ion and that there is a single binding site (n). With the exception of Fe(II), all metal binding affinity measurement were performed using enzyme concentrations lower than K_d_ in order to ensure an equilibrium driven situation.

The titration data for metal stoichiometry (n) determination were analyzed by nonlinear curve fitting using Eq (3) [35], [36], [37]. This equation was also used to calculate K_d_ for Fe(II) by converting fluorescence intensity into percent saturation. Since the enzyme concentration used is higher relative to the estimated K_d_ for Fe(II), Eq (3) was used for determining both stoichiometry (n) and K_d_ for Fe(II).

$$f=f_{o}+(f_{m}-f_{o})\frac{(nP+x+K_{d})-\sqrt[{2}]{{\left(nP+x+K_{d}\right)}^{2}-4nPx}}{2nP}$$ (3)

where, f is the fluorescence signal resulting from binding by the metal cofactor, f_o_ is the signal from a blank enzyme in solution when no metal was bound, f_m_ corresponds to the maximal quenched fluorescence intensity when metal was bound, K_d_ is the dissociation constant, P, x are total protein and added metal ion concentrations respectively, n is the number of binding sites (stoichiometry).

## Results

### Expression and purification of DddW

The *dddW* gene from *R*. *pomeroyi* was amplified by PCR and cloned into pET16b. The recombinant DddW protein was expressed in *E*. *coli* as an N-terminal His-tagged fusion and purified as soluble protein with a yield of 12–15 mg per liter of bacterial culture and 95% purity as indicated by SDS-PAGE gel (S1 Fig and S1 Table). DddW, as-isolated from *E*. *coli* cells grown in LB media consistently contained 0.2–0.4 equivalents of bound iron when analyzed for metal content. All trace of Fe was removed from DddW to form apo-DddW by the addition of the metal chelator EDTA and confirmed by ICP-OES analysis. This apo-DddW was used in all subsequent studies. Significantly, when assayed for DMSP lyase activity the as-isolated DddW gave 0.28 μmol s^-1^ mg^-1^ whereas apo-DddW activity was barely detectable (0.02 μmol s^-1^ mg^-1^), thus confirming an important role for a metal ion as a cofactor in DddW. The molecular mass of the denatured enzyme was in the range of 18–22 kDa and the predicted molecular mass of His-tagged protein based on amino acid sequence is 18 kDa. Size exclusion chromatographic analysis of DddW reveals predominantly a 36 kDa species, which compared to standards indicate a dimeric protein (S2 Fig). A molecular mass of 35 kDa was determined by light scattering studies, thereby confirming that the enzyme exists as a dimer in solution. The secondary structure of DddW was determined by circular dichroism (CD) and the far-UV spectrum exhibits a single shallow minimum between 210–220 nm, which suggests that the secondary structure in solution is primarily beta-sheet as observed in cupin superfamily proteins, including DddQ [25], [31], [32] (S3 Fig).

### Metal ion uptake of DddW

Having determined that DddW requires a metal cofactor for DMSP lyase activity and that as isolated from *E*. *coli* shows the presence of iron, we tested the favored incorporation of metal cofactors into DddW. We found that the expression of recombinant DddW in minimal media in the absence of any added metal ion led to the formation of insoluble protein. However, when DddW was grown in minimal media in the presence of a mixture of metal ions in equal quantities consisting of Mn(II), Fe(III), Co(II), Ni(II), Cu(II), and Zn(II), soluble DddW protein was produced, further supporting the importance of metal cofactors for proper folding of the protein and solubilization. Soluble DddW protein was purified to homogeneity and metal analysis of the isolated protein showed that although other metals were present iron was clearly preferentially bound (S2 Table). The data suggests that the cupin-containing lyase DddW can accommodate multiple metal ions, but the preferred metal ion is iron as it is primarily sequestered from the growth media despite its lower position in the Irving Williams Series.

### Metal ion binding affinity and stoichiometry studies

**Fig 3:**
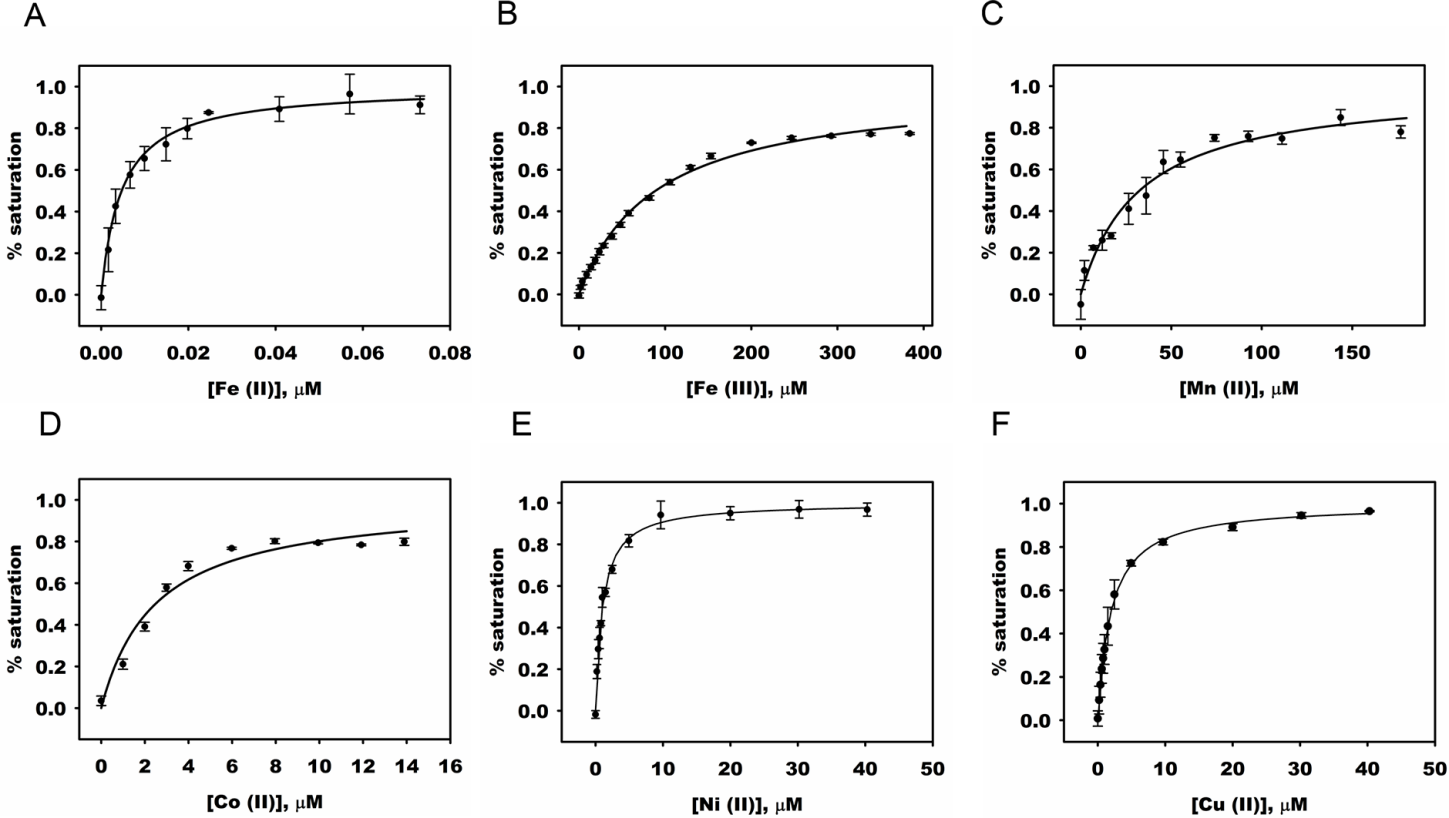
Metal binding affinities (K_d_) of DddW. Titration of apo-DddW with increasing concentrations of the metal ions Fe(NH_4_)_2_(SO_4_)_2_ (added anaerobically), FeCl_3_, CoCl_2_, MnCl_2_, CuCl_2_ or NiCl_2_ was done. The binding was monitored by saturation of the fluorescence intensities. The concentration of enzyme used is as follows: (A) 0.5 μM apo-DddW, (B)-(F) 2 μM apo-DddW. The K_d_ values were: (A) Fe(II), 4.7 ± 0.0 nM; (B) Fe(III), 89.3 ± 4.3 μM; (C) Mn(II), 32.7 ± 5.0 μM; (D) Co(II), 2.5 ± 0.4 μM; (E) Ni(II), 1.0 ± 0.1 μM; (F) Cu(II), 1.9 ± 0.2 μM.

To determine metal ion binding affinity, fluorescence quenching experiments were performed using apo-DddW in the presence of various metal ions. Fluorescence studies are commonly used to identify metal-binding, stoichiometry, and selectivity of metal ions as has been shown previously [35], [38]. The quenching of intrinsic tryptophan fluorescence signal in DddW was utilized as an indicator of metal binding when a metal ion was added to a solution of apo-DddW. Based on our homology model of DddW (Fig 2B), Trp51 is suspected to be in close proximity to the active site allowing us to perform fluorescence quenching studies. By measuring percent quenching with increasing concentration of metal ion, the binding affinities of DddW for Mn(II), Fe(III), Co(II), Ni(II), Cu(II), and Zn(II) were determined by fitting the data to Eq (2) or Eq (3) for Fe(II) (Fig 3). DddW has the highest affinity for binding Fe(II) at 5 nM, followed by Co(II), Ni(II), and Cu(II) at ~ 1–3 μM, with Mn(II) and Fe(III) exhibiting weaker binding affinities of 33 μM and 89 μM respectively. These data indicate that while the enzyme is promiscuous in its metal recognition, it strongly favors binding to Fe(II).

**Fig 4:**
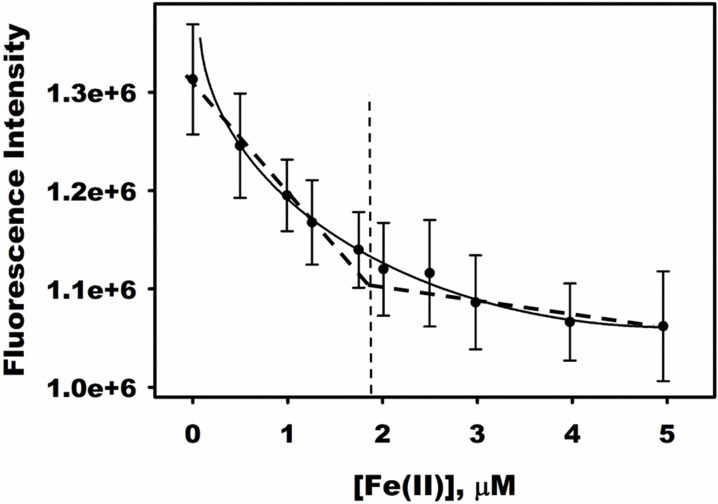
Stoichiometry of Fe(II) binding to DddW. 2 μM apo-DddW (under tight-binding conditions) was titrated with increasing concentrations of Fe(NH_4_)_2_(SO_4_)_2_ and the fluorescence intensity was monitored. The titration data were analyzed by nonlinear curve fitting using Eq (3) to produce the solid line. Upon data fitting, the stoichiometric ratio of Fe(II) to DddW monomer was determined to be 1:1.

The metal ion stoichiometry was determined by monitoring quenching of fluorescence intensity in the presence of Fe(II). An enzyme concentration sufficiently higher than the K_d_ was used to approach a tight-binding condition [35], [36], [39]. The plot of decreasing fluorescence intensity as a function of increasing concentration of Fe(II) added to apo-DddW showed an inflection point where the stoichiometric ratio of Fe(II) to DddW monomer was determined to be 1:1 (Fig 4).

### Detection of lyase reaction products by MS

To reaffirm that DddW catalyzes the cleavage of DMSP to produce acrylate and DMS, enzymatic assays were analyzed by LC-MS. The components that eluted around 14 mins corresponding to the acrylate peak were subjected to ESI. The spectrum contained only one major peak below a molecular weight of 100 g/mol measured at m/z 71.0094, calculated for M^-^ ion of 71.0133 (S4 Fig). This peak confirms the formation of acrylate indicating that DddW is a DMSP-lyase as reported [34]. Residual DMSP was not detected in a measurable amount in any of the elution fractions. Dimethyl sulfide formed during the lyase reaction was detected by gas chromatography.

### Catalytic Properties of DddW

**Fig 5:**
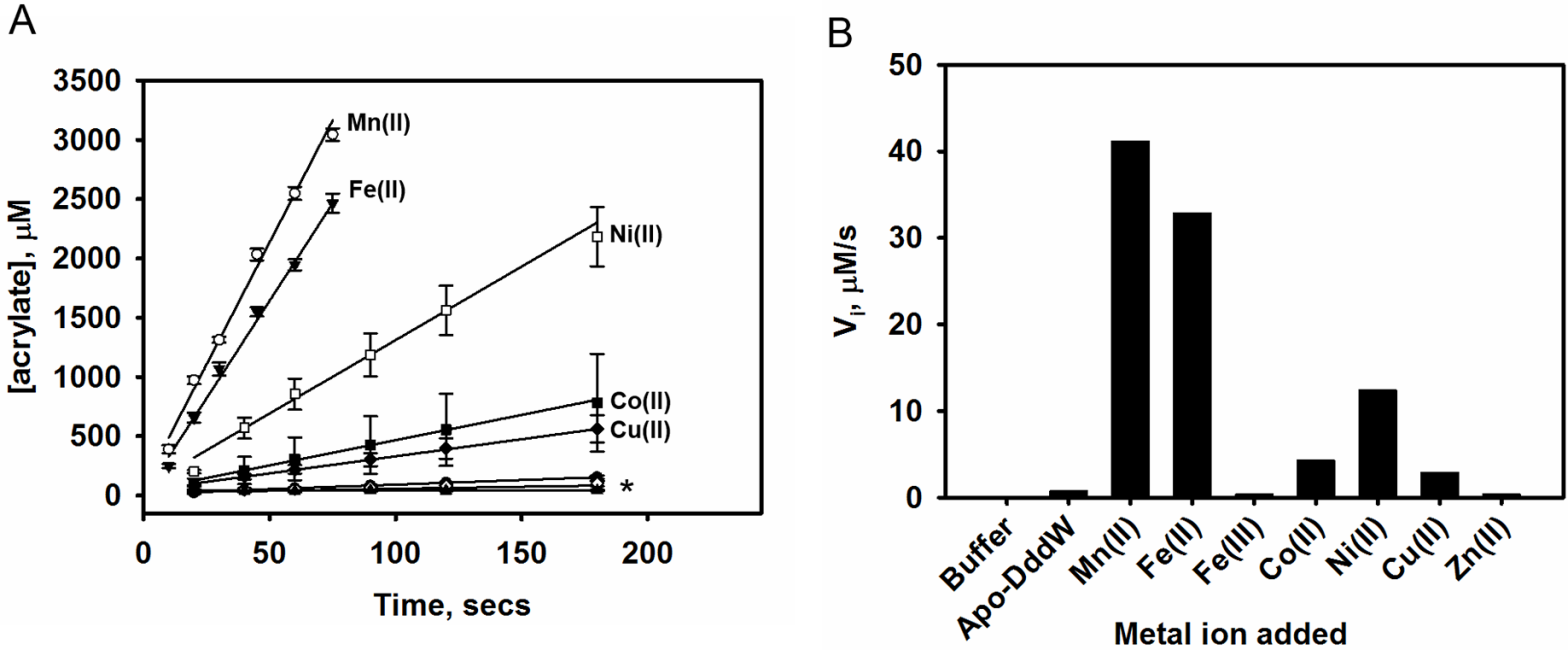
Effects of various transition metal ions on the DMSP lyase activity of DddW. All metal ions were added so that they were at least 90% bound (the calculations were based on the K_d_ of individual metal ions). Assays were performed anaerobically at pH 8.0 and room temperature. The activity of Apo-DddW without metal ions was used as a reference (100%). (A) Determination of the initial velocity (V_i_) of DddW catalyzed reaction in presence of various transition metal ions: Mn(II), Fe(II), Fe(III), Co(II), Ni(II), Cu(II), and Zn(II). For clarity, plots of Fe(III), Zn (II), apo-DddW, and buffer control are indicated by ‘*’. (B) Effect of metal ions on the initial velocity (V_i_) of DddW catalyzed lyase reaction.

The catalytic activity of DddW was tested anaerobically in the presence of DMSP and various metal ions and the formation of the acrylate product was monitored by HPLC. The reaction buffer was used as a background control and the lyase activities were measured relative to apo-enzyme (Fig 5A and 5B). The presence of Fe(II) or Mn(II) elicited a significant increase (~40-fold) in DddW lyase activity compared to the apo-enzyme. DddW activity was also enhanced to a lesser extent by Co(II), Ni(II), and Cu(II), but Zn(II) and Fe(III) had a slight inhibitory effect. The inhibitory effect in the presence of Fe(III) is mostly due to the lack of solubility of Fe(III) at higher concentrations at pH 8.0 causing precipitation [40].

**Fig 6:**
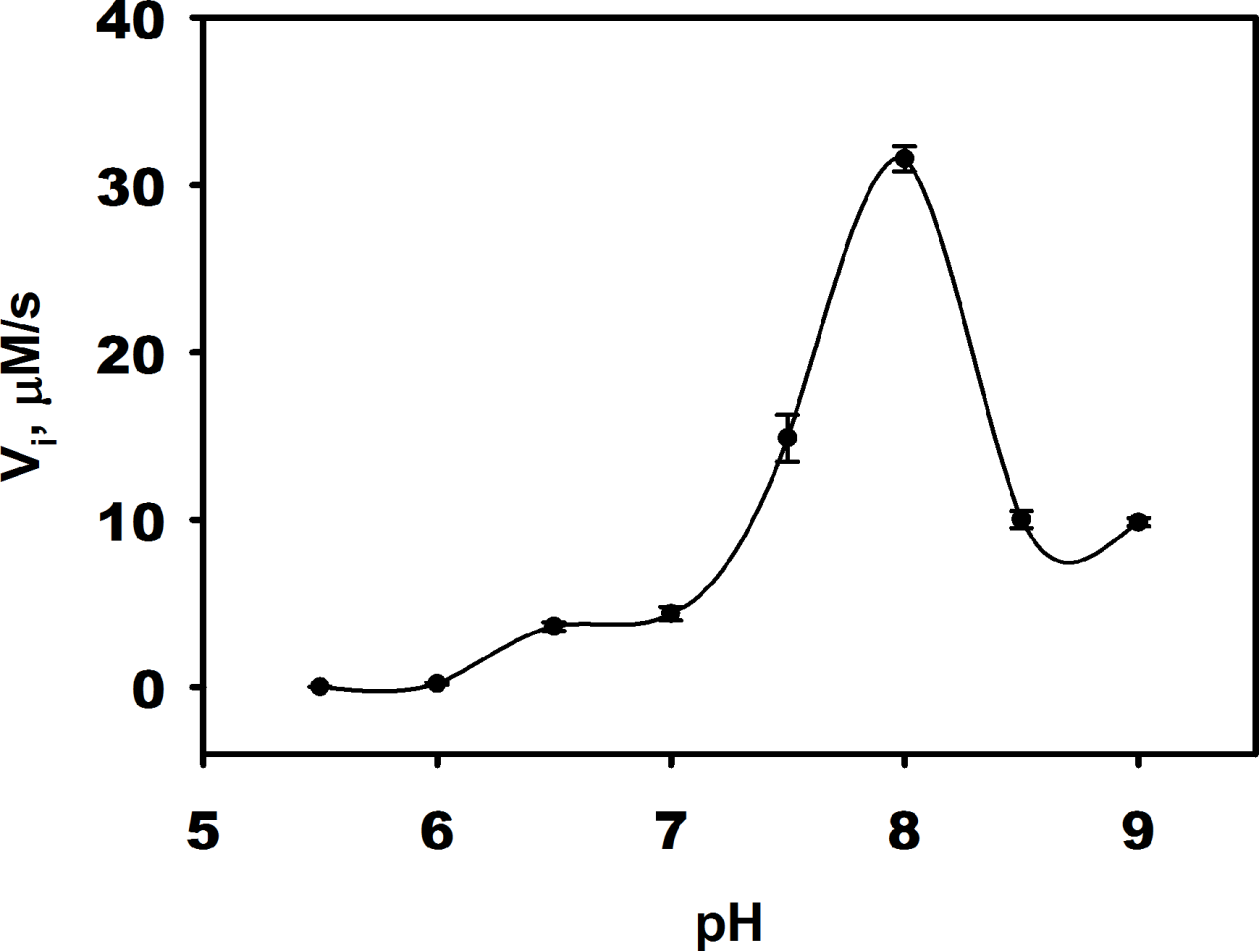
The pH dependence of DddW lyase activity. The optimal pH was determined by comparing the initial velocities (V_i_) of reactions containing 2 μM apo-DddW, 2 μM Fe(II), and 10 mM DMSP in varying buffer solutions. The buffers used are as follows: 50 mM MES 20 mM NaCl (pH 5.5, 6.0, 6.5), 50 mM HEPES 20 mM NaCl (pH 7.0, 7.5, 8.0), 50 mM Tris-HCl 20 mM NaCl (pH 8.5, 9.0).

The pH dependence of DddW activity was measured at pH values between 5.5 and 9.0 using saturating concentrations of DMSP (10 mM) in the presence of Fe(II). The maximum activity (32 μM s^−1^) of DddW was observed at pH 8.0 (Fig 6), which is the same as that observed for DddQ [25]. The optimal activities of several lyases and peptidases have been reported to be between pH 7.0 and 9.0 [41–45]. Because the p*K*_*a*_ values for carboxylates are 2–5, the fully deprotonated form of DMSP will be bound at the optimal pH for DddW.

**Fig 7:**
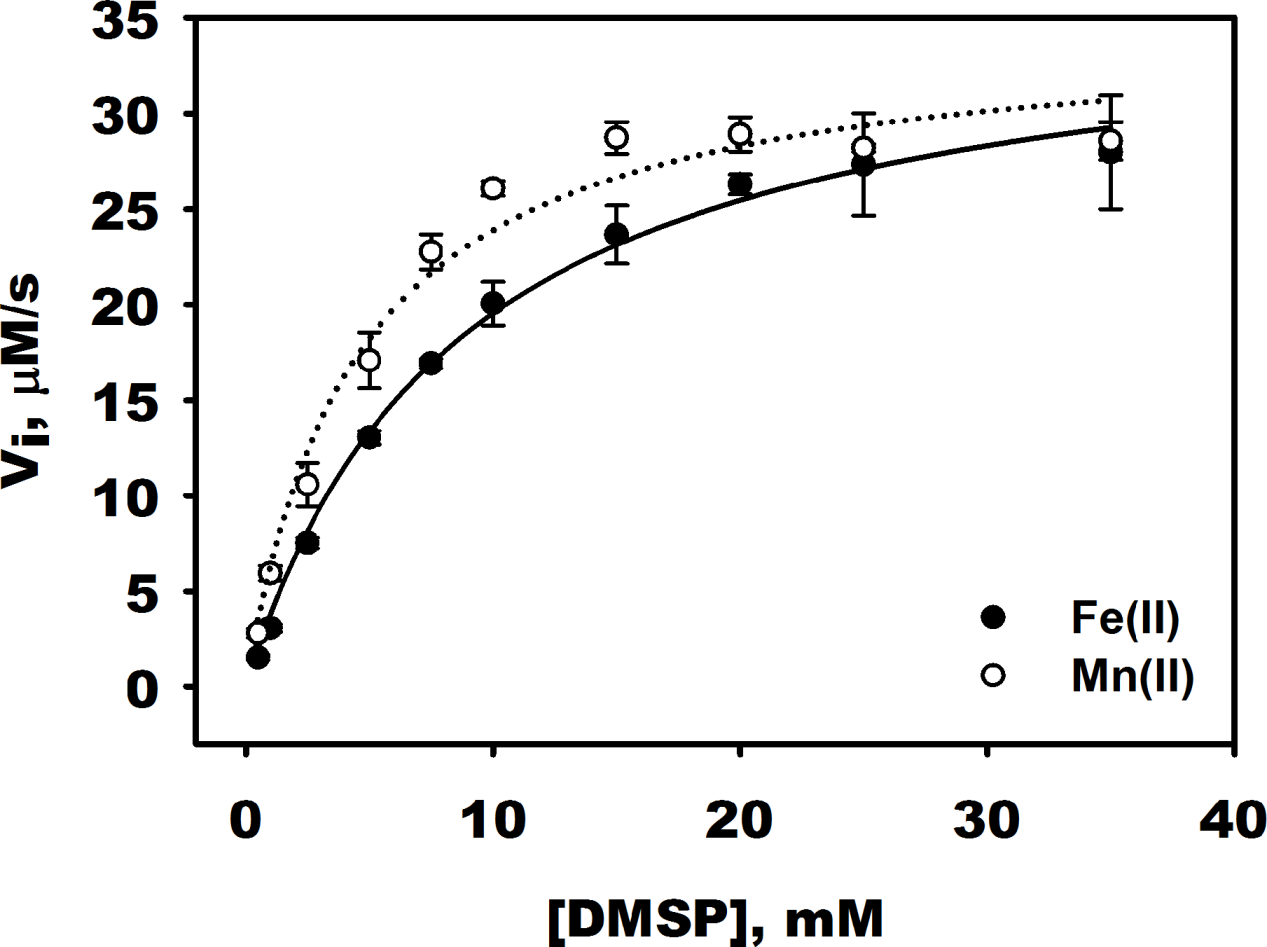
Dependence of initial velocity (V_i_) of DddW catalyzed lyase reaction on DMSP concentrations in the presence of Fe(II) and Mn(II). Apo-DddW (2 μM) was mixed with an equimolar amount of Fe(NH_4_)_2_(SO_4_)_2_ and 300μM MnCl_2_. To this reaction mixture, varying concentrations (0.5–35 mM) of DMSP was added. The reactions were monitored at 205 nm. The data were fit to the Michaelis-Menten equation. The kinetic parameters are as follows. With Fe(II): V_max_ = 36.50 ± 1.27 μM/s; k_cat_ = 18.25 s^-1^; *K*_m_ = 8.68 ± 0.73 mM; *k*_cat_/*K*_m_ = 2.10 x 10^3^ M^-1^s^-1^; With Mn(II): V_max_ = 34.66 ± 1.64 μM/s; k_cat_ = 17.33 s^-1^; *K*_m_ = 4.50 ± 0.75 mM; *k*_cat_/*K*_m_ = 3.85x10^3^ M^-1^s^-1^.

As maximal lyase activity was observed with Fe(II) and Mn(II), the kinetic parameters for the DddW catalyzed elimination reaction were determined in the presence of Fe(II) and Mn (II) by varying DMSP concentrations over the range of 0.5 mM to 35 mM (Fig 7). Homodimeric DddW catalyzed the elimination of DMSP using Fe(II) with a specific activity of 1.08 μmol s^-1^ mg^-1^, *k*_cat_ of 18.25 s^−1^ at pH 8.0, a *K*_*m*_ for DMSP of 8.68 ± 0.73 mM, and K_cat_/K_m_ of 2.10 x10^3^ M^-1^s^-1^. In the presence of Mn(II) the specific activity was 1.02 μmol s^-1^ mg^-1^, *k*_cat_ of 17.33 s^−1^ at pH 8.0, a *K*_*m*_ for DMSP of 4.50 ± 0.75 mM, and K_cat_/K_m_ of 3.85 x10^3^ M^-1^s^-1^. Although these K_m_ values appear high for an enzyme that specifically cleaves DMSP, they are similar to those published for the DMSP lyase DddP and that of the DMSP demethylase DmdA [27], [46].

The above results together clearly suggest that DddW is a mononuclear iron-dependent enzyme. Since many mononuclear iron enzymes utilize dioxygen for catalysis, we sought to assess if DddW is an oxidase. The oxygen dependence of activity was tested in the presence of Fe(II) and O_2_ and the enzymatic reaction showed no evidence of molecular oxygen utilization during the lyase reaction leading to DMS production.

### Spectral Characterization of DddW

**Fig 8:**
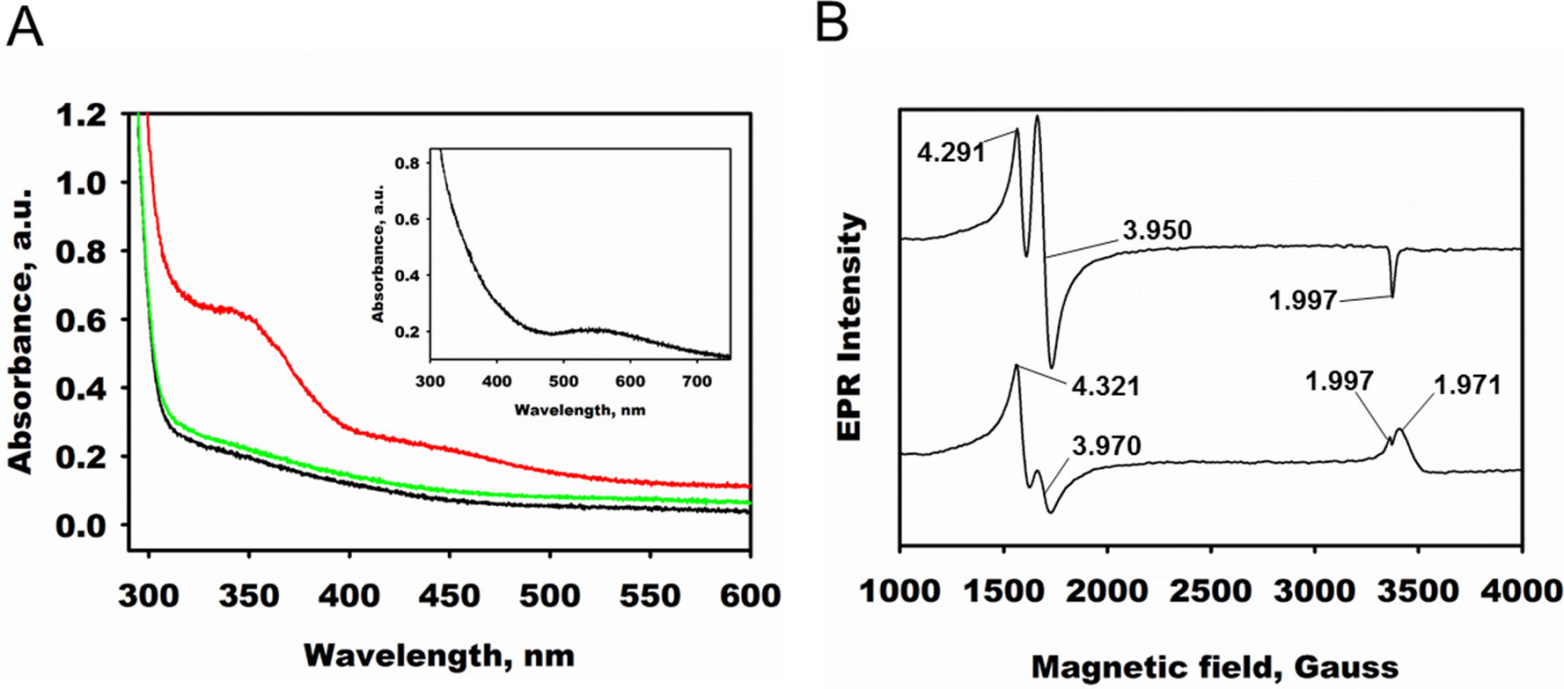
Spectral properties of Fe(II)-bound DddW. (A) UV-visible spectra of the reaction of as-isolated DddW in the presence of Fe(II) and Cu(II). All spectra with Fe(II) had an enzyme concentration of 370 μM. Trace in black, apo-DddW, green, apo-DddW in presence of 370 μM Fe(II) red, apo-DddW+Fe(II) after bubbling with NO gas. The absorption maximum is at 340 nm with a shoulder at 430 nm. Inset: Spectrum of 1 mM apo-DddW in the presence of Cu(II). The spectral feature at 550 nm is due to a charge transfer transition of DddW with Cu(II). (B) EPR spectra of: (top) 18 μM apo-DddW with Fe(II); (bottom) Fe(II)-DddW in the presence of 25 mM DMSP. The spectra were collected at microwave frequency, 9.43 GHz; receiver gain, 2 x 104; modulation frequency, 100 kHz; temperature, 4 K; microwave power, 200 microwatts; 83.89 s sweep time, and 16 scans.

In order to gain insight into the mechanism of DddW and other DMSP lyases, UV-visible and EPR spectroscopic studies were performed with the catalytic active preferred metal ion, Fe(II). The UV-visible spectrum of apo-DddW exhibited no absorption features above 280 nm. The addition of Fe(II) to apo-DddW also had no effect on the absorption spectrum. However, the absorbance in the 300–500 nm region developed a broad shoulder when the Fe(II)-bound DddW protein was exposed to NO (Fig 8A). Addition of NO to the reduced Fe(II) enzyme resulted in a yellow complex with an absorption maximum of 340 nm and a second spectral feature near 430 nm. Similar spectral changes were observed when the iron complex of isopenicillin N synthase was saturated with NO [47]. To assess whether the binding of a catalytically less competent cofactor caused a change in absorption properties of DddW, UV-visible spectral studies were carried out in the presence of Cu(II). A new spectral feature arose at 550 nm when Cu(II) was added to a concentrated solution of apo-DddW (Fig 8A, inset). This feature is due to a charge transfer transition of the Cu(II)-DddW complex and had also been observed for Cu-Zn superoxide dismutase and a copper dependent laccase, SLAC upon Cu(II) binding [48], [49].

EPR studies of Fe(II)-bound DddW in the presence of NO shows an EPR active species with resonances at g = 4.291, 3.950, 1.997 (Fig 8B, top). These spectral features are characteristic of species with spin S = 3/2, which is consistent with the nitrosyl complexes seen in other mononuclear Fe(II) containing proteins [47], [50]. In contrast, apo-DddW with reduced Fe(II) exists in a low-spin state and is EPR silent. Anaerobic addition of DMSP to the reduced Fe(II)-DddW-NO complex resulted in an EPR active species with a spectrum distinct from that of Fe(II)-DddW-NO (Fig 8B, bottom). Perturbation of the EPR signal indicates that substrate does not prevent NO binding and instead its presence alters the electronic environment around the Fe(II) center. The spectrum due to Fe(II)-DddW-NO-DMSP complex was observed regardless of the order of addition of substrate and NO to Fe(II) loaded DddW, suggesting an open coordination site around iron (through either monodentate substrate binding or bidentate substrate) allowing NO binding.

### DddW variants (H81A, H83A, E87A, H121A)

**Fig 9:**
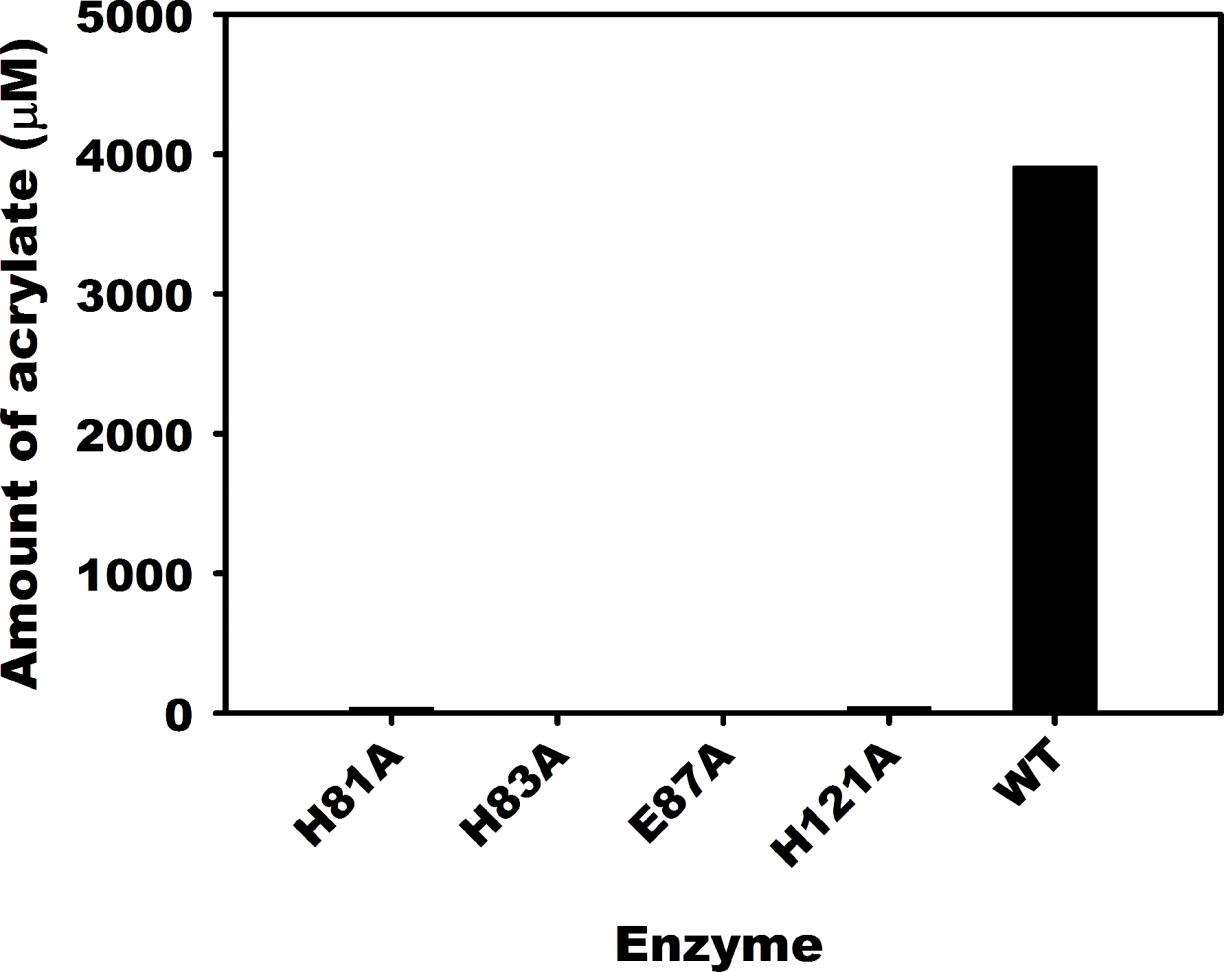
DMSP lyase activity of DddW variants compared to wild-type enzyme. Activity assays were performed anaerobically using 2 μM DddW, 2 μM Fe(NH_4_)_2_(SO_4_)_2_ and 10 mM DMSP. The reactions were incubated for 15 mins before quenching.

From the results described, it is evident that DddW is a metalloenzyme and preferentially requires Fe(II) for catalysis. To determine the residues required for metal binding and catalysis, alanine variants of suspected metal-binding residues, H81A, H83A, E87A, and H121A, were investigated. These residues are contained and conserved within the cupin domain of DddW that is expected to constitute the metal-binding active site (Fig 2A). The DddW variants were purified from *E*. *coli* cells grown in LB media, as described above for wild type DddW (S5A Fig). After metal analysis we found that DddW variant enzymes contained 10-fold less iron per molecule relative to wild-type DddW indicating a significant reduction in their capacity to bind their metal cofactor (S5B Fig). To test whether these DddW variants that were severely reduced in their ability to bind iron were affected in their DMSP lyase activity, we incubated purified apo-DddW variants (after EDTA treatment for removal of any residual iron) with Fe(II) and DMSP for 15 minutes and then assayed for acrylate production. HPLC analysis revealed that none of the DddW mutants formed the acrylate product compared to the wild-type enzyme that was active (Fig 9), thereby indicating that His81, His83, Glu87, and His121 are necessary for iron binding and for DMSP lyase activity of DddW.

## Discussion

DMSP is a key nutrient in marine environments and its catabolism *via* the DMSP lyase pathway generates DMS [5]. DMS is an important nutrient for bacteria [10], but when released into the atmosphere it provides ~60% of global biogenic sulfur flux to the atmosphere [7], [51] and forms cloud condensation nuclei of importance in climate and the global sulfur cycle [13], [52]. Recent molecular studies have uncovered the diversity of DMSP lyases with six different Ddd enzymes (DddL, DddP, DddQ, DddY, DddW, and DddY) of distinct protein families being identified in wide-ranging bacteria and fungi [5]. DddD is an atypical DMSP lyase that generates 3-hydroxypropionate and DMS from DMSP [5], [23]. The other known Ddd enzymes, are carbon-sulfur lyases that degrade DMSP to DMS, acrylate and a proton. Despite the recent biochemical and mechanistic advances in our understanding of the DddD, DddP, DddQ enzymes [23], [14], [26], there are still many DMSP lyases left unexplored at the biochemical level.

One such DMSP lyase previously unstudied at the biochemical level is DddW that was isolated in the model roseobacter *R*. *pomeroyi*. Roseobacters are abundant marine bacteria that can account for 30% of bacteria in coastal regions and ~3% of cells in the open ocean surface waters [53], [54]. Significantly, they have been shown to dominate in many DMSP-rich marine environments [55], [56]. Like many Roseobacters [5] *R*. *pomeroyi* can both demethylate DMSP, containing the DMSP demethylase DmdA, and lyse DMSP having three functional DMSP lyases (DddP, DddQ, and DddW). Of these enzymes DddW is the major DMSP induced gene product (40-fold) in *R*. *pomeroyi* with the *dddP* and *dddQ* genes being only modestly up-regulated (1–4 fold) [57]. Despite its likely importance in *R*. *pomeroyi*, DddW is only present in two other sequenced roseobacters *Roseobacter* sp. MED193 and *Pseudophaeobacter arcticus*. Although DddW is not abundant in marine bacteria it does contains a C-terminal cupin domain (Fig 2A) and is representational of other cupin containing DMSP lyase enzymes, including DddL and DddQ, the latter of which occurs more frequently in marine roseobacters and has multiple representatives in nearly all the metagenomic Global Ocean Sampling (GOS) sampling sites [5]. It is of interest to compare findings on the biochemical characterization of DddW to those made in the recently reported zinc-bound structure of DddQ where a mechanism of DMSP cleavage has been proposed [25].

Here we report the first example of a metal-dependent DMSP lyase in which more than one metal ion can catalyze an elimination reaction of the substrate likely utilizing a 3-His-1-Glu metal-binding catalytic motif. No definitive experiments were presented in [25] to demonstrate that DddQ requires metals or if the Zn-bound form is active since metal ions were not stripped from the as-purified DddQ protein. It was shown that as-purified DddQ lyase activity was enhanced by the addition of Mn(II) and Co(II) which is consistent with our studies on DddW. Our studies support the predicted cupin residues of DddW (H81, H83, E87 and H121) binding to metal ions and contributing to the catalytic activity. These key metal-binding residues in DddW are conserved in the other known cupin-containing DMSP lyases DddL and DddQ, and in the latter have been substituted to alanines and shown to significantly decrease DddQ DMSP lyase activity, in line with our findings. Although we have not determined the crystal structure of DddW showing the exact metal-binding site comprising of the 3-His-1-Glu center, an overlay of a homology model of DddW (generated based on structures of various other cupin domain containing proteins [58]) on DddQ structure illustrates the close proximity of these residues and the probable metal binding site (Fig 2B).

Whilst the mechanism of DMSP lyase by DddW is not established here we have demonstrated DddW requires metals for structural integrity and proper folding of the protein. *E*.*coli* cells expressing DddW in minimal media lacking any metal ion, led to insoluble protein, but the addition of selective transition metal ions during protein expression led to the incorporation of the corresponding metal ion resulting in soluble protein. Also, when challenged with a mixture of metal ions, all the metal ions were incorporated into the enzyme at various ratios, but with iron clearly preferred. This demonstrates the promiscuity of DddW for metal ions and that Fe(II) is its cofactor of choice, something exemplified by the fact that the as-isolated DMSP lyase expressed in *E*.*coli* has a high affinity for iron and preferentially sequesters iron from the growth media. Such promiscuity also exists with DddQ that accepts different metal ions even though it was isolated with the inhibitory zinc ion [25]. Such promiscuity likely also exits with the M24 metallopeptidase DMSP lyase DddP enzyme that was isolated with a mixture of different metal ions, but the structure shows two iron ions [26].

Having established that DddW is a metalloenzyme with a preference for Fe(II) we determined the *in vitro* metal binding properties of DddW using tryptophan fluorescence quenching in the presence of various divalent transition metal ions. DddW binds these metal ions with varying affinities (Fig 3). The binding affinity of the enzyme for tested transition metal ions follows the order: Fe(II)>Ni(II),Cu(II)>Co(II)>Mn(II)>Fe(III), with that Fe(II) having a K_d_ 213-fold lower than Ni(II). The titration of Zn(II) to DddW reduced the tryptophan fluorescence quenching efficiency and resulted in an increase in fluorescence intensity. Such an increase in fluorescence is known to result from the interaction of histidine and cysteine residues with added zinc [39], [59]. Thus, these binding studies confirm that DddW binds different metal ions with varying sizes, but strongly favors Fe(II). Indeed, similar fluorescence characterization of other cupin containing enzymes, e.g. Dke1 dioxygenase show metal promiscuity but a strong preference for Fe(II) [39].

For the DddW catalyzed DMSP lyase reaction, Fe(II) and Mn(II) were the most effective metals showing similar V_i_ rates despite Mn(II) binding being ~200 fold weaker than Fe(II) (Figs 3, 5, and 7). The severely reduced lyase activity with Fe(III), to levels below that of apo-DddW, is mostly likely due to solubility issues associated with higher concentrations of Fe(III) at pH 8.0. Transition metal ions such as, Co(II), Ni(II),and Cu(II) showed reduced activity compared to Fe(II) and Mn(II), which can be attributed to a change in metal coordination geometry from six-coordinate with Fe(II) and Mn(II) to five- or four-coordinate with Co(II), Ni(II), and Cu(II) and/or substrate positioning. Both of these factors could place DMSP in a less active conformation. Since the carboxylate terminus of DMSP can bind to a metal ion in either a monodentate or bidendate fashion, a sterically and conformational favorable binding mode will probably be the determining factor for tuning the catalytic activity. The monodentate binding of DMSP has been observed with an alanine mutant of DddQ [25]. The binding and selectivity of metal ions in other metalloproteins such as phosphoserine phosphatase and ribonucleotide reductase R2 are known to effect carboxylate binding mode, which in turn affects enzyme activity [60]. The almost complete lack of activity in the presence of Zn(II) is most likely due to the fact that Zn(II) favors a tetrahedral geometry and the four metal coordination sites could be occupied by the four active site residues (3-His-1-Glu), thus not allowing DMSP to bind. Activity measurements with the four-coordinate Zn(II)-bound DddQ suggests that Zn(II) inhibits the enzyme activity [25]. These data together suggest that Fe(II) is the preferred catalytic metal ion and in the absence of iron, DddW can bind to other metal ions. Moreover, it is apparent that metal ions likely have important roles in protein stability and enzyme activity in cupin containing (DddL, DddQ, and DddW) and metallopeptidase family DMSP lyases [25], [26], [27]. It will be of interest to see if DddY, yet to be biochemically characterized, also requires a metal co-factor for enzyme activity like these other CoA independent ‘typical’ DMSP lyases.

In contrast to Fe(II), DMSP is loosely bound to DddW as reflected in the relatively high K_m_ value of 8.68 mM (Fig 7). However, the K_m_ value is very similar to those published for the DMSP lyase DddP (13.8 mM) and DMSP demethylase DmdA (5.4 mM) also from *R*. *pomeroyi* [27], [46]. *R*. *pomeroyi* is known to accumulate high intracellular concentrations of DMSP (70 mM), likely using it as an osmoprotectant [46]. Thus, the presence of DMSP lyase and demethylase enzymes with high K_m_ values for their DMSP substrate may be an adaptation by marine bacteria that utilize DMSP as an osmoprotectant allowing them to only catabolize DMSP, which is in excess of their cellular requirement. It is striking that DddY of *Alcaligenes* (1.4 mM), *Desulfovibrio acrylicus* (0.45 mM), and DddD of *Marinomonas* MWYL1 (67 μM), with much lower K_m_ values than DddW, DddP, and DmdA, are from organisms that either grow on DMSP as a sole carbon source or that use the acrylate generated from DMSP as electron acceptor for anaerobic respiration [21], [23], [61], [62], [63]. Thus, it is possible that the function of DMSP in the organism containing it governs the efficiency of the DMSP catabolic enzymes binding to DMSP as to ensure its cellular DMSP requirement are satisfied.

The EPR spectrum of Fe(II)-DddW yielded no noticeable signal. This is expected because reduced Fe(II) has all six valence electrons paired in a low-spin state. In the presence of nitric oxide (NO), a high-spin state is induced giving rise to an EPR signal (Fig 8B, top). Since NO coordinates to a metal ion, this data implies the presence of a bound iron because NO would not bind if a metal ion was not present at the active site. The binding of NO also indicates that there is an open coordination site around iron to accommodate the substrate. If all the iron sites were bound by protein ligands, it suggests that there would be no room for substrate DMSP binding, strongly supporting iron as the active metal for DddW. In addition, we predict that NO binds to the Fe(II) center by displacing a loosely bound water from the active site of DddW. When substrate is added to the reduced Fe(II)-enzyme-NO complex a change in the spectrum is observed due to a change in the electronic environment around the Fe(II) center (Fig 8B, bottom). If DMSP binds in a bidentate fashion when NO is present, it would indicate that the Fe(II) center is bound to three protein residues; however, it is possible for the iron cofactor to be bound to four residues and only bind DMSP in a monodentate fashion.

**Fig 10:**
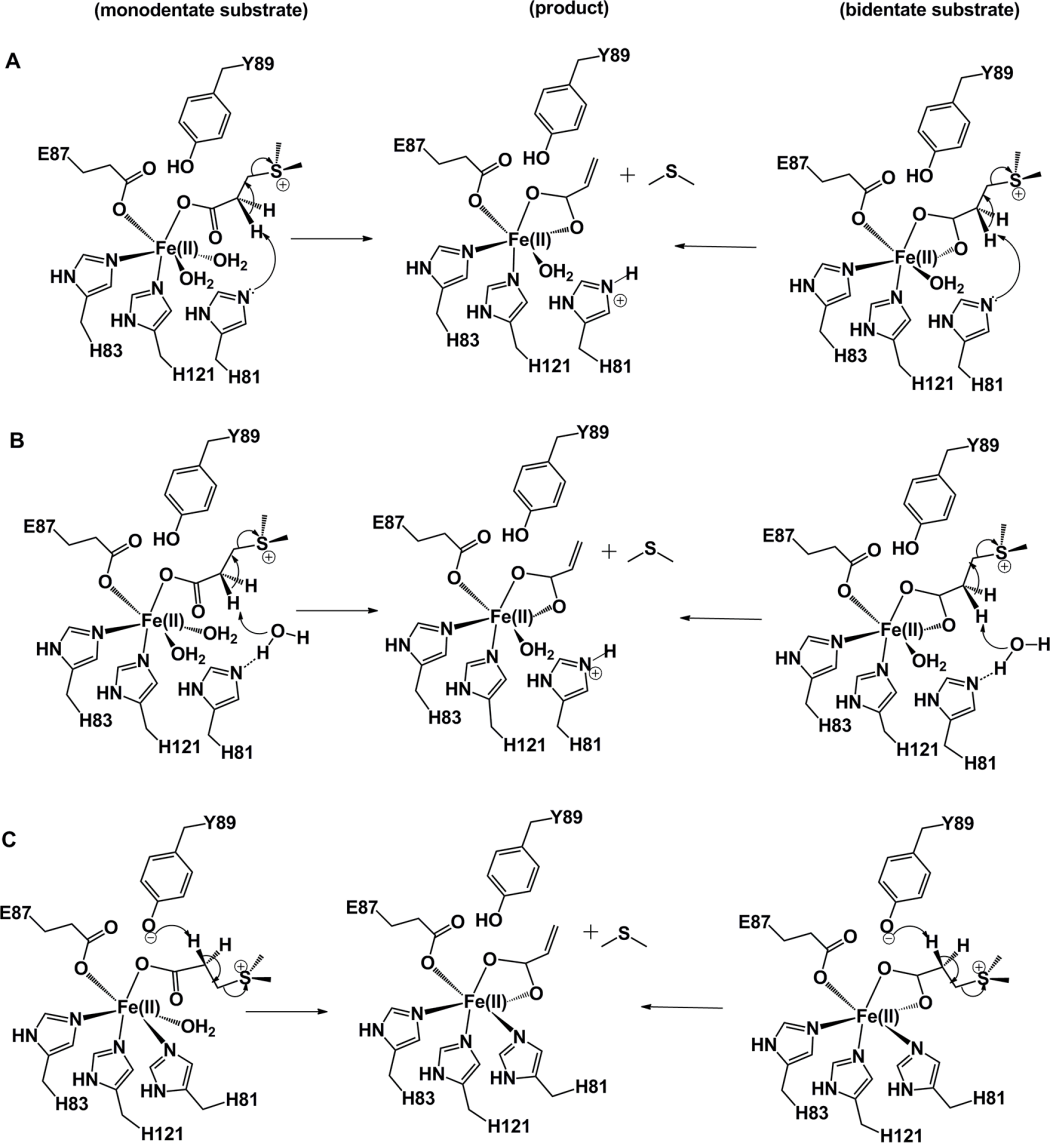
Proposed mechanisms for the mononuclear iron dependent DMSP lyase, DddW. DddW binds to Fe(II) cofactor to which the substrate can coordinate in either monodentate or bidentate modes. (A) His81 can act as a nucleophile to remove a hydrogen atom from the α-carbon of DMSP to form acrylate. (B) A hypothetical water molecule can be activated by His81, which then acts as a nucleophile in initiating catalysis. (C) Tyr89 located near the active site can initiate the elimination reaction cleaving DMSP.

Based on these studies, a mechanism of DddW-catalyzed reaction is proposed, wherein the metal center would possibly be coordinated by 3His-1-Glu ligands (H81, H83, E87, and H121) and the substrate can bind either in monodentate or bidentate mode, with the remaining coordination sphere around Fe(II) occupied by water molecules (Fig 10). Significantly, when each of these four potential ligands are replaced by an alanine individually the resulting DddW variants showed decreased iron content and complete loss of DMSP lyase activity, thus suggesting that all four residues (H81, H83, E87, and H121) are involved in substrate positioning, iron binding, and/or catalysis. A basic histidine can act as a nucleophile to remove a hydrogen atom from the α-carbon of DMSP to form acrylate (Fig 10A). Alternatively, a hypothetical water molecule can be activated by histidine, which then acts as a nucleophile in abstracting the hydrogen atom resulting in DMSP cleavage (Fig 10B). An overlay of a homology model of DddW on the Zn-bound DddQ structure shows the catalytic residues H81, H83, E87, and H121 in close proximity to the metal binding site (Fig 2B). The proposed metal binding residues of DddW (H83, E87, and H121) superimpose well on the zinc-coordinating DddQ residues (H125, E129, and H163). While Tyr usually is not involved in metal ion binding in cupin proteins, the DddQ structure shows a Zn-coordinated Tyr residue (Tyr131) that is proposed to initiate catalysis [25] and this Tyr aligns well on Tyr89 of DddW (Fig 2B). Thus, the possible involvement of a tyrosine (Tyr89) in DddW acting as a base to initiate the elimination reaction cannot be ruled out (Fig 10C).

The results presented here show for the first time that DddW is metal dependent, catalyzing the beta-elimination of DMSP releasing acrylate and volatile DMS as products, thus adding to the repertoire of the metalloenzymes of the cupin superfamily. Furthermore, a metal-binding site and relevant catalytic residues have been identified thereby confirming that the residues His81, His83, Glu87, and His121 are required for the lyase activity of DddW. In future studies, it is important to probe the mechanism by studying substrate analogs/inhibitors, determining the X-ray crystal structure of DddW to identify the metal binding residues and substrate positioning, and to further explore site-directed mutagenesis strategies, which would allow identification of the molecular determinants of metal-binding and catalysis.

## Supporting Information

S1 Fig SDS-PAGE gel of DddW purification. Lanes labeled as: whole cell lysate (WCL), cell free lysate (CFL), flow through (FT), wash sample, and elution fractions 1–10. MWM: molecular weight marker.
(TIF)

S2 Fig Size-exclusion chromatographic analysis of DddW. The peak that elutes near 35 mL corresponds to an approximate molecular weight of 36 kDa, which corresponds to the DddW dimer. The peak that elutes around 25 mL corresponds to a molecular weight of about 170 kDa, which indicates some aggregation of the enzyme.
(TIF)

S3 Fig CD spectra of wild-type apo-DddW. A shallow single dip between 210–220 nm indicates β-sheet secondary structure.
(TIF)

S4 Fig Product identification by LC-MS. The lyase reaction was performed with 2 μM each of apo-DddW and Fe(NH_4_)_2_(SO_4_)_2_ in the presence of 10 mM DMSP. Mass spectrum showing the peak at 71.0094 corresponds to the acrylate product.
(TIF)

S5 Fig Metal analysis of DddW mutants. (A) SDS-PAGE gel of purified enzymes, WT, H81A, H83A, E87A, H121A, isolated by growing in LB media. (B) ICP-OES of EDTA treated DddW mutants compared to that of wild-type DddW, indicating loss of Fe content in the mutant proteins.
(TIF)

S1 Table DddW activity and yield during the course of purification. Whole cell lysate (WCL), cell free lysate (CFL), post Ni-NTA, post size exclusion (post SEC), and after a freeze-thaw.
(PDF)

S2 Table DddW preferentially uptakes iron from growth media. Metal analysis using ICP-OES of wild-type apo-DddW grown in minimal media supplemented with a mixture of chloride salts of metal ions, Mn(II), Co(II), Fe(III), Ni(II), Cu(II), and Zn(II).
(PDF)
