# Supplementary figures and images for: Biochemical, Kinetic, and Spectroscopic Characterization of Ruegeria pomeroyi DddW—A Mononuclear Iron-Dependent DMSP Lyase
Source: PLoS One. 2015 May 19;10(5):e0127288. doi: 10.1371/journal.pone.0127288 (PMC4437653; doi:10.1371/journal.pone.0127288)

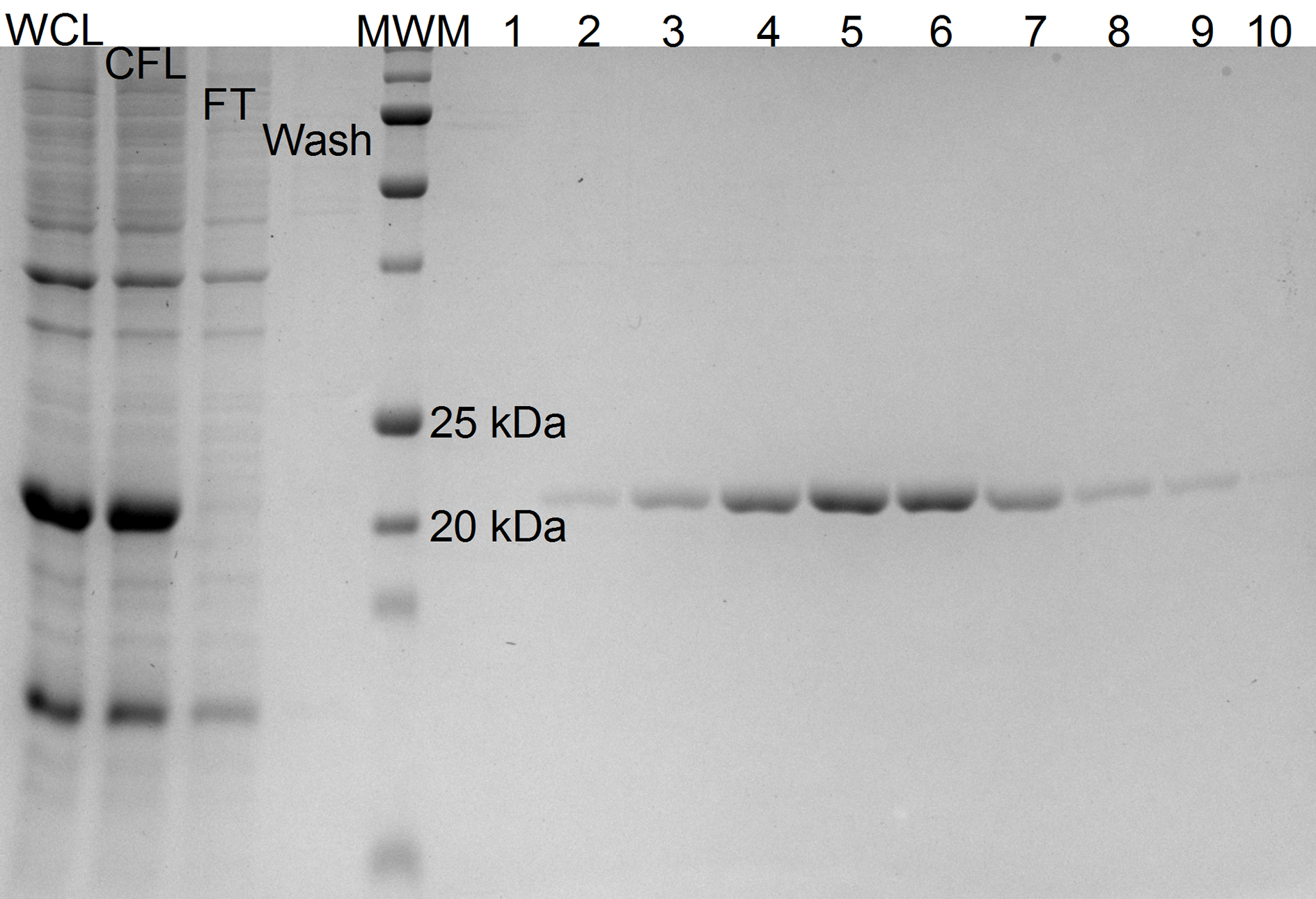

Supplement: S1 Fig — Lanes labeled as: whole cell lysate (WCL), cell free lysate (CFL), flow through (FT), wash sample, and elution fractions 1–10. MWM: molecular weight marker. (TIF) [file pone.0127288.s001.tif]

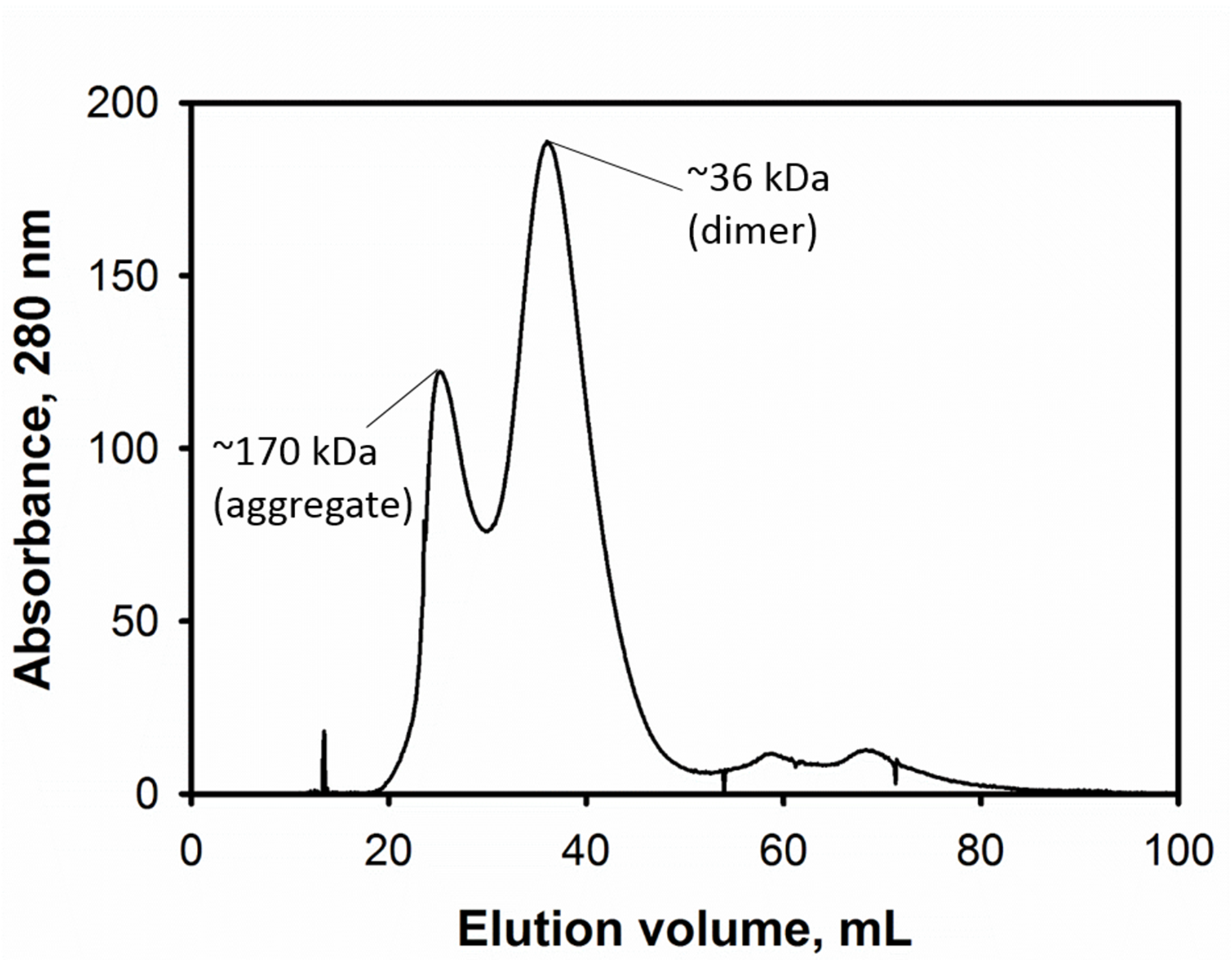

Supplement: S2 Fig — The peak that elutes near 35 mL corresponds to an approximate molecular weight of 36 kDa, which corresponds to the DddW dimer. The peak that elutes around 25 mL corresponds to a molecular weight of about 170 kDa, which indicates some aggregation of the enzyme. (TIF) [file pone.0127288.s002.tif]

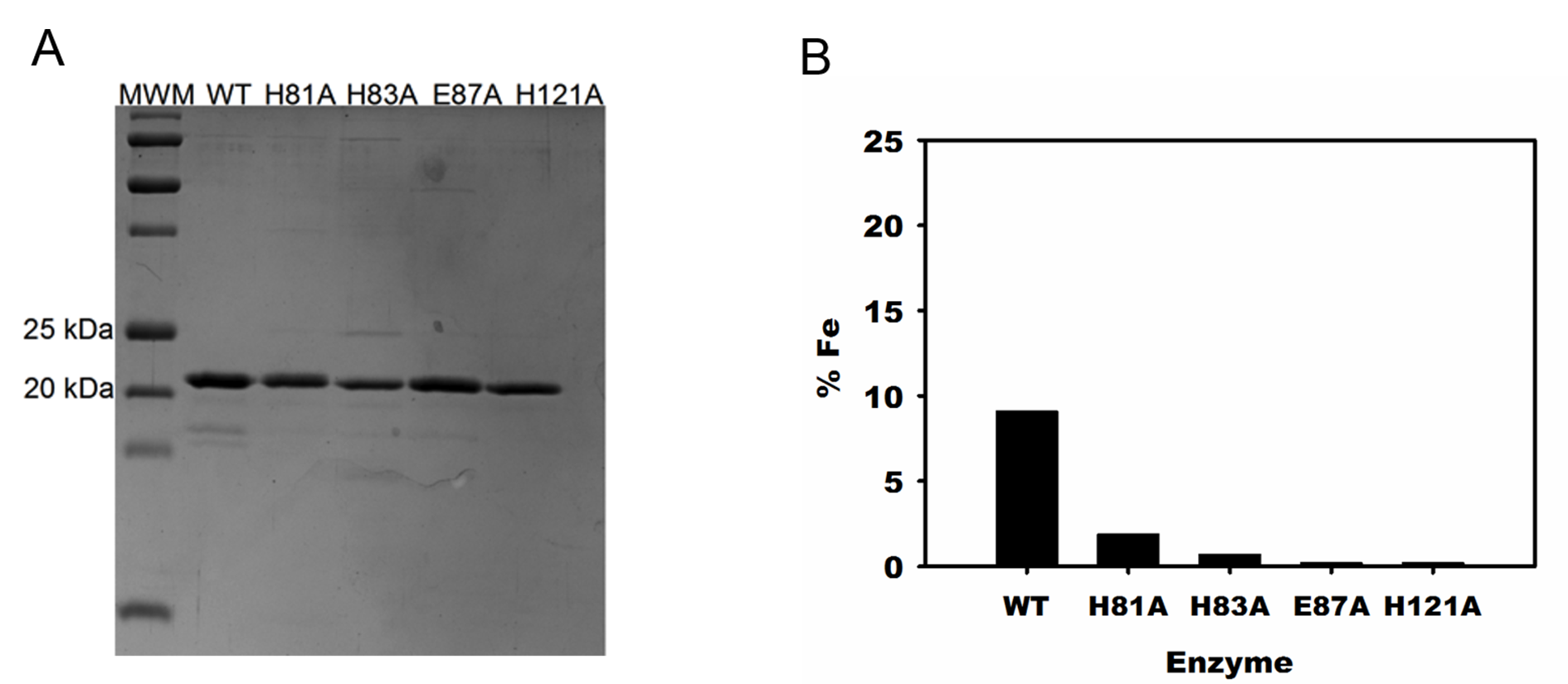

Supplement: S5 Fig — (A) SDS-PAGE gel of purified enzymes, WT, H81A, H83A, E87A, H121A, isolated by growing in LB media. (B) ICP-OES of EDTA treated DddW mutants compared to that of wild-type DddW, indicating loss of Fe content in the mutant proteins. (TIF) [file pone.0127288.s005.tif]
