# Supplementary material for: Biochemical, Kinetic, and Spectroscopic Characterization of Ruegeria pomeroyi DddW—A Mononuclear Iron-Dependent DMSP Lyase
Source: PLoS One. 2015 May 19;10(5):e0127288. doi: 10.1371/journal.pone.0127288 (PMC4437653; doi:10.1371/journal.pone.0127288)
Supplement: S1 Table — Whole cell lysate (WCL), cell free lysate (CFL), post Ni-NTA, post size exclusion (post SEC), and after a freeze-thaw. (PDF) [file pone.0127288.s006.pdf]

Table S1. **DddW activity and yield during the course of purification**. Whole cell lysate (WCL), cell free lysate (CFL), post Ni-NTA, post size exclusion (post SEC), and after a freeze-thaw.

	**Total volume (mL)**	**Concentration (mg/mL)**	**Total protein (mg)**	**Activity (µM of acrylate)**	**Total Activity (µmol of acrylate)**	**Specific Activity (µmol/(sec*mg))**	**Purification factor**	**Yield (%)**
WCL	30	139.36	4180.8	5049.80	151.49	0.01	1	100
CFL	25	113.98	2849.5	5267.28	131.68	0.02	1.53	87
post Ni-NTA	60	0.63	37.8	1833.27	110.00	0.40	40.15	73
post SEC	5.75	3.52	20.2	8122.58	46.70	3.34	332.25	31
freeze thaw	5.75	3.52	20.2	7703.46	44.29	3.17	-	-
