# Supplementary material for: Biochemical, Kinetic, and Spectroscopic Characterization of Ruegeria pomeroyi DddW—A Mononuclear Iron-Dependent DMSP Lyase
Source: PLoS One. 2015 May 19;10(5):e0127288. doi: 10.1371/journal.pone.0127288 (PMC4437653; doi:10.1371/journal.pone.0127288)
Supplement: S2 Table — Metal analysis using ICP-OES of wild-type apo-DddW grown in minimal media supplemented with a mixture of chloride salts of metal ions, Mn(II), Co(II), Fe(III), Ni(II), Cu(II), and Zn(II). (PDF) [file pone.0127288.s007.pdf]

Table S2. **DddW preferentially uptakes iron from growth media**. Metal analysis using ICP-OES of wild-type apo-DddW grown in minimal media supplemented with a mixture of chloride salts of metal ions, Mn(II), Co(II), Fe(III), Ni(II), Cu(II), and Zn(II).

**Metal content (mol metal ion/mol of DddW)**						
	Mn	Fe	Co	Ni	Cu	Zn
DddW	0.050	0.208	0.068	0.072	0.021	0.108
